# Neural Engagement During a Video-Based Theory of Mind Task: A Pilot fMRI Study in Schizophrenia

**DOI:** 10.1192/j.eurpsy.2026.10515

**Published:** 2026-07-31

**Authors:** I. París-Gómez, B. Yildirim, A. Serra-Mayoral, N. Ramiro-Sousa, P. Del Olmo, A. Aquino-Servin, S. García-Rojas, S. Leonangeli, S. Vargas, P. J. Mckenna, P. Fuentes-Claramonte, E. Pomarol-Clotet

**Affiliations:** 1 FIDMAG Research Foundation; 2 Fundació Hospitalàries Barcelona Nord; 3 Fundació Hospitalàries Barcelona; 4 Fundació Hospitalàries Sant Boi; 5 CIBERSAM ISCIII, Barcelona, Spain

## Abstract

**Introduction:**

Theory of Mind (ToM) - the capacity to infer others’ mental states – is consistently impaired in patients with schizophrenia (SZ), and is a well-established predictor of social dysfunction and poor functional outcomes. While numerous functional imaging studies have examined ToM in SZ, few have employed videos, which may be important for ecological validity and a more nuanced understanding of social cognitive deficits in the disorder.

**Objectives:**

This pilot study aimed to: 1) validate a novel video-based task for engaging the core ToM neural network, and 2) analyse differences in brain activation between SZ patients and healthy controls (HC).

**Methods:**

15 HC and 6 SZ patients matched for sex, age and estimated premorbid IQ underwent fMRI while performing a video-based ToM task adapted from the Battery for Theory of Mind Assessment (BAT). The task included 16 videos, 8 showing social situations with a ToM component, and 8 control videos featuring people performing various non-social activities, each followed by a question (either ToM-related or related to a concrete aspect of the scene) (Figure 1). Data were analysed using FSL’s FEAT with a GLM approach (cluster-based threshold: z > 2.6, p < .05) to compare brain activity while responding to ToM relative to control questions.

**Results:**

Behavioral performance was significantly worse in the SZ group compared to HCs on the ToM questions (p < .05). Both groups activated the canonical ToM network during the task, including the medial prefrontal cortex (mPFC) and the precuneus (both nodes of the default mode network), and the temporoparietal junction (TPJ). The comparison between patients and controls revealed four clusters of significant hyperactivation in the dorsomedial prefrontal cortex (dmPFC), precuneus/posterior cingulate cortex, the right inferior parietal lobule and the cerebellum (Figure 2).

**Image 1:**
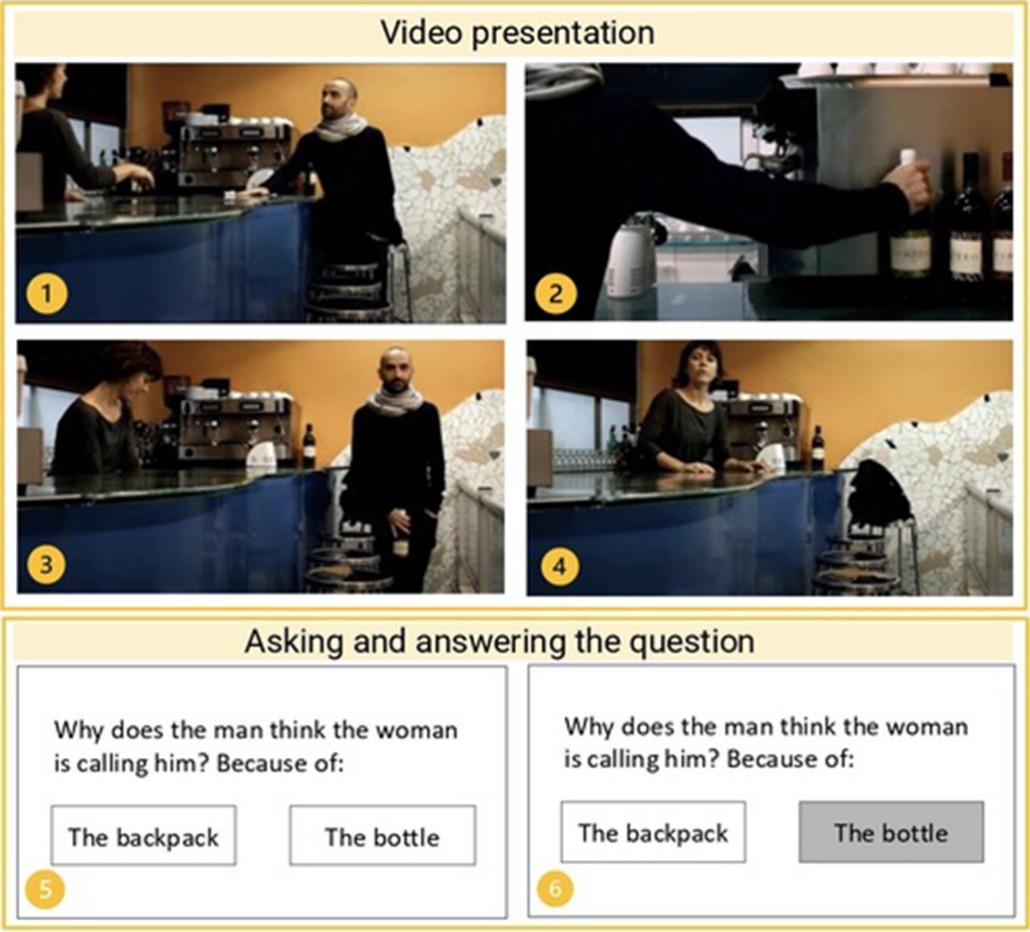
Image 1: Long description.

**Image 2:**
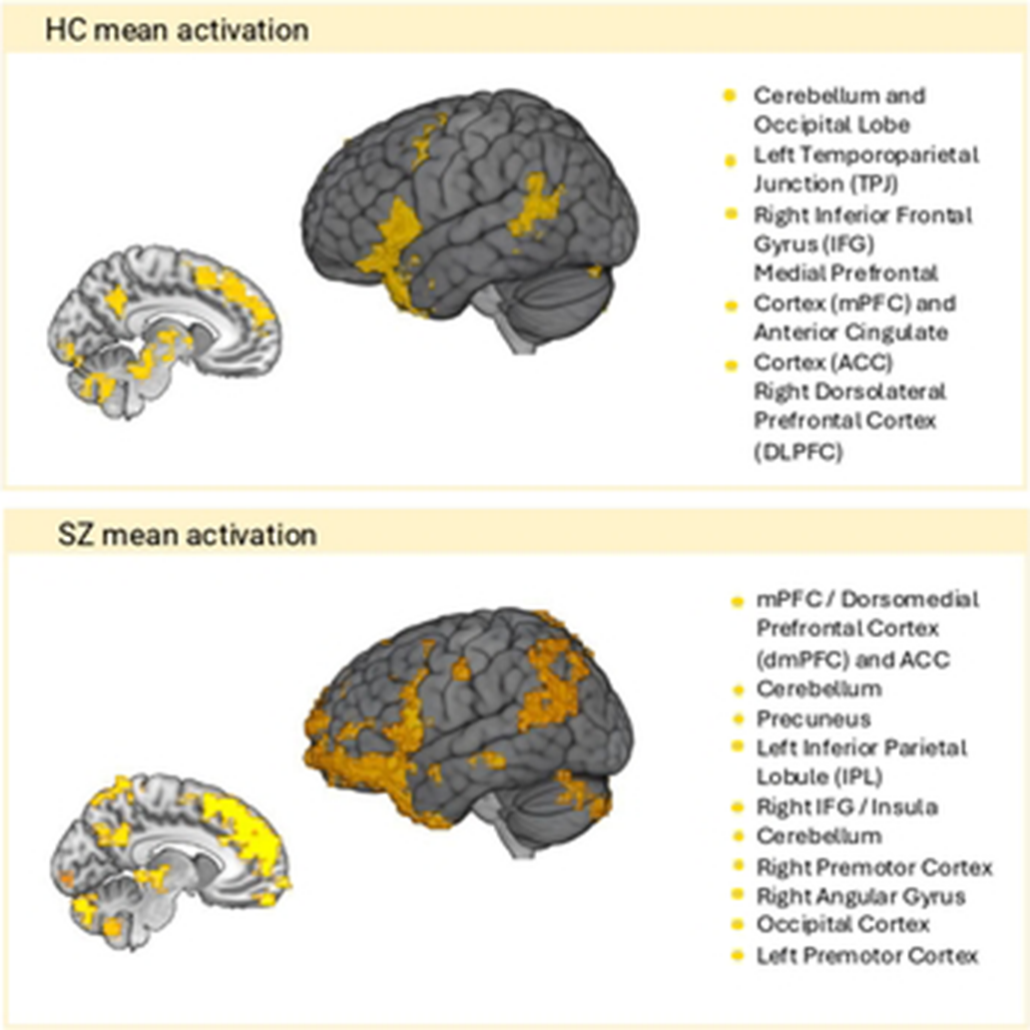
Image 2: Long description.

**Conclusions:**

This video-based task robustly engaged the canonical ToM network in both healthy subjects and SZ patients, supporting its utility for studying the neural correlates of ToM. Our results for the comparison between patients and controls are preliminary, but point to potential hyperactivation in parts of the default mode network in SZ patients.

**Disclosure of Interest:**

None Declared

## Long descriptions

### Image 1: Long description

The figure is divided into two main sections.

The top section, titled Video presentation, contains four panels numbered 1 to 4.

* Panel 1: A man with a scarf stands behind a blue curved bar in a cafe while a woman stands on the opposite side.

* Panel 2: A close-up shows a hand reaching for a white-capped bottle among other wine bottles on a shelf.

* Panel 3: The man stands behind the bar holding the bottle while the woman looks down at the counter.

* Panel 4: The woman looks up toward the man while he is partially obscured by a black backpack placed on a stool.

The bottom section, titled Asking and answering the question, contains two panels numbered 5 and 6.

* Panel 5: Displays the question, Why does the man think the woman is calling him? Because of: followed by two white buttons labeled The backpack and The bottle.

* Panel 6: Displays the same question and buttons, but the button for The bottle is highlighted in gray, indicating it is the selected answer.

Navigate back to Image 1:.

### Image 2: Long description

The figure consists of two horizontal panels.

Top Panel: H C mean activation.

On the left is a medial sagittal view of the brain showing yellow activation clusters in the cerebellum and medial prefrontal regions.

In the center is a lateral view of the right hemisphere showing activation in the temporal and frontal lobes.

On the right, a bulleted list identifies the active regions:

* Cerebellum and Occipital Lobe

* Left Temporoparietal Junction (T P J)

* Right Inferior Frontal Gyrus (I F G)

* Medial Prefrontal Cortex (m P F C) and Anterior Cingulate Cortex (A C C)

* Right Dorsolateral Prefrontal Cortex (D L P F C).

Bottom Panel: S Z mean activation.

On the left is a medial sagittal view showing more extensive yellow activation across the superior frontal and parietal regions compared to the top panel.

In the center is a lateral view showing widespread activation across the frontal, parietal, and temporal lobes.

On the right, a bulleted list identifies the active regions:

* m P F C / Dorsomedial Prefrontal Cortex (dm P F C) and A C C

* Cerebellum

* Precuneus

* Left Inferior Parietal Lobule (I P L)

* Right I F G / Insula

* Cerebellum

* Right Premotor Cortex

* Right Angular Gyrus

* Occipital Cortex

* Left Premotor Cortex.

Navigate back to Image 2:.

